# Inhibition of ABL1 by tyrosine kinase inhibitors leads to a downregulation of MLH1 by Hsp70-mediated lysosomal protein degradation

**DOI:** 10.3389/fgene.2022.940073

**Published:** 2022-10-20

**Authors:** Hannah G. Daniels, Breanna G. Knicely, Anna Kristin Miller, Ana Thompson, Rina Plattner, Eva M. Goellner

**Affiliations:** ^1^ University of Kentucky, College of Medicine Department of Toxicology and Cancer Biology, Lexington, KY, United States; ^2^ Berea College, Berea, KY, United States; ^3^ University of Kentucky Markey Cancer Center, Lexington, KY, United States; ^4^ University of Kentucky, College of Medicine Department of Pharmacology and Nutritional Sciences, Lexington, KY, United States

**Keywords:** mismatch repair, nilotinib dasatinib, DNA repair, Hsp70, lysosome

## Abstract

The DNA mismatch repair (MMR) pathway and its regulation are critical for genomic stability. Mismatch repair (MMR) follows replication and repairs misincorporated bases and small insertions or deletions that are not recognized and removed by the proofreading polymerase. Cells deficient in MMR exhibit an increased overall mutation rate and increased expansion and contraction of short repeat sequences in the genome termed microsatellite instability (MSI). MSI is often a clinical measure of genome stability in tumors and is used to determine the course of treatment. MMR is also critical for inducing apoptosis after alkylation damage from environmental agents or DNA-damaging chemotherapy. MLH1 is essential for MMR, and loss or mutation of MLH1 leads to defective MMR, increased mutation frequency, and MSI. In this study, we report that tyrosine kinase inhibitors, imatinib and nilotinib, lead to decreased MLH1 protein expression but not decreased MLH1 mRNA levels. Of the seven cellular targets of Imatinib and nilotinib, we show that silencing of ABL1 also reduces MLH1 protein expression. Treatment with tyrosine kinase inhibitors or silencing of ABL1 results in decreased apoptosis after treatment with alkylating agents, suggesting the level of MLH1 reduction is sufficient to disrupt MMR function. We also report MLH1 is tyrosine phosphorylated by ABL1. We demonstrate that MLH1 downregulation by ABL1 knockdown or inhibition requires chaperone protein Hsp70 and that MLH1 degradation can be abolished with the lysosomal inhibitor bafilomycin. Taken together, we propose that ABL1 prevents MLH1 from being targeted for degradation by the chaperone Hsp70 and that in the absence of ABL1 activity at least a portion of MLH1 is degraded through the lysosome. This study represents an advance in understanding MMR pathway regulation and has important clinical implications as MMR status is used in the clinic to inform patient treatment, including the use of immunotherapy.

## Introduction

DNA mismatch repair (MMR) is the repair process that repairs small insertions and deletions, and base/base mispairs generated and not corrected by the replicative DNA polymerase during DNA replication (Li, 2008; Fishel, 2015). This correction of replication errors by MMR is necessary for genomic stability, and MMR deficiency leads to increased mutation rates and genomic instability (Kolodner, 1995; Gupta and Heinen, 2019). MMR defects and the resulting genomic instability promote cancer development, specifically colorectal and endometrial cancers (Borresen et al., 1995; de la Chapelle, 2004; Boland and Goel, 2010; Kastrinos and Stoffel, 2013; Lynch et al., 2015). Proteins in the MMR pathway also play various roles outside of repairing replication errors. These include preventing recombination between divergent sequences (Spies and Fishel, 2015; Tham et al., 2016) and promoting apoptosis in the presence of exogenously generated mispairs that are recognized by but not repaired by MMR, such as those formed after damage by alkylating agents or platinum agents (Jiricny, 2006; Topping et al., 2009; Fu et al., 2012; Gupta and Heinen, 2019). Loss of mismatch repair results in resistance to these classes of chemotherapeutics (Modrich and Lahue, 1996). MMR defects have recently been positively correlated with increased response to immunotherapy, presumably due to their increased mutational burden and neoantigen production (Kubecek et al., 2016; Zhao et al., 2019).

Canonical eukaryotic MMR involves the recognition of the mispair by MutSα, a heteroduplex consisting of MSH2 and MSH6, followed by DNA nicking and excision licensing of the mispair after recruitment of MutLα, a heteroduplex consisting of endonuclease MLH1 and PMS2. Exonuclease 1 (EXO1) is then recruited to excise the mispair, or this step is carried out by one of the more recently elucidated EXO1-independent subpathways (Goellner et al., 2015; Calil et al., 2021). Finally, the replicative DNA polymerases, PCNA, and RFC carry out DNA synthesis to fill in the gap, and ligase completes repair (Bowen et al., 2013; Goellner et al., 2015; Bowen and Kolodner, 2017). EXO1-independent MMR can proceed through either multiple rounds of MLH1-PMS2 nicking (Amin et al., 2001; Smith et al., 2013; Goellner et al., 2014) or FEN1 activity can compensate during the excision step (Calil et al., 2021).

Exogenously induced mispairs can occur when damaged bases, such as O^6^ methylguanine generated by S_N_1 DNA alkylating agents, become preferentially paired with an incorrect base by the replicative polymerases (Li et al., 2016). Repair of O^6^-methylguanine lesion is usually performed by direct reversal by methylguanine methyltransferase (MGMT); however, the absence of or deficiencies in MGMT lead to unrepaired lesions, which upon DNA replication can generate O^6^meG/T mispairs (Kaina et al., 2007). This mispair is then a substrate for MMR recognition. While these damage induced mispairs are recognized by the MMR machinery, they are ultimately unable to be repaired as the damage is on the template strand and therefore result in signaling the cell for apoptosis (Meikrantz et al., 1998; Stojic et al., 2004; Li et al., 2016). Studies have shown that the components of MutSα and MutLα heteroduplexes are required for this DNA damage response (Dosch et al., 1998; Cejka et al., 2003). Loss of either of these critical MMR components leads to resistance to alkylation-induced apoptosis.

Due to the importance of MMR in the maintenance of genomic stability, the regulation of the MMR response pathway is of particular interest. Regulation of the MutSα complex has been shown in several studies to have cell cycle-dependent transcriptional regulation of MSH2 and MSH6 (Edelbrock et al., 2013; O'Brien and Brown, 2006; Seifert and Reichrath, 2006). Studies have shown that transcriptional regulation involving hypermethylation of the MLH1 promoter relates directly to MMR deficiency in tumors (Esteller et al., 1998; Niv, 2007). Additionally, mechanisms such as phosphorylation and protein degradation have been suggested to elicit posttranslational regulation of MLH1 and PMS2 (Jia et al., 2016; Weßbecher and Brieger, 2018). Phosphorylation of the MutLα heteroduplex has primarily been shown to occur on serine/threonine residues by various kinases (Jia et al., 2016; Hinrichsen et al., 2017b; Weßbecher and Brieger, 2018; Weßbecher et al., 2018). Degradation of the MutLα proteins often occurs due to complex instability caused by mutations in one of the heteroduplex partners, as MLH1 and PMS2 are thought to be obligate heterodimers, and heteroduplex formation is required for activity (Mohd et al., 2006; Abildgaard et al., 2019). Additionally, PCNA phosphorylation has been shown to control MMR activity (Ortega et al., 2015; Tong et al., 2015).

In this study, we show that MLH1 levels are decreased by tyrosine kinase inhibitor, Dasatinib, and the more specific and clinically relevant tyrosine kinase inhibitors, imatinib and nilotinib. This effect was observed in both non-cancerous HEK293 cells and in cells derived from colorectal human tumors. Further, we show that of the shared Dasatinib and imatinib/nilotinib targets, knockdown of the ABL1 kinase by siRNA also decreases MLH1 protein expression. This decrease in expression is sufficient to disrupt MMR function, as cells treated with tyrosine kinase inhibitors or Abl siRNA became partially resistant to alkylation-induced apoptosis. This regulation of MLH1 by Abl kinase and tyrosine kinase inhibitors is posttranslational as mRNA levels remain steady after treatment, and we observe phosphorylation of MLH1 that is lost with ABL1 inhibition. We propose that Abl prevents MLH1 from being targeted for degradation by the chaperone HSP70 and that in the absence of ABL1 activity, at least a portion of MLH1 is degraded through the lysosome. Together this study elucidates a new mechanism of MMR regulation. Furthermore, as many tyrosine kinase inhibitors targeting ABL1 are approved for clinical use, this study has interesting implications on the potential side effects on MMR in cancers typically treated with tyrosine kinase inhibitors. Additionally, this study opens possibilities of clinically modulating MMR for enhanced response to immunotherapy.

## Materials and methods

### Chemicals and reagents

Imatinib mesylate (imatinib) was obtained from Tocris Biosciences; 10 mM stock was prepared in dimethylsulfoxide (DMSO) (Fisher Scientific) and stored at −20°C. Nilotinib was obtained from Cell Signalling Inc.; 10 mM stock was prepared in DMSO and stored at −20°C. Dasatinib was obtained from Cell Signalling Inc.; 10 mM stock was prepared in DMSO and stored at -20°C. 6-Thioguanine 98% (6 TG) was obtained from TCI America (T0212-1G); 40 mM stock was prepared in NaOH and stored at −20°C. Methylnitronitrosoguanidine (MNNG) was obtained from Sigma-Aldrich (Cat #129941); 10 mM stock was prepared in DMSO and stored at −20°C. Antibodies used in this study include MLH1 (#3515, #4256), PCNA (#2586, #13110), ABL1 (#2862), and Phospho-Tyrosine MultiMab (#8954) (Cell Signalling Inc.), IRDye 800CW Donkey anti-Rabbit IgG Secondary Antibody and IRDye 800CW Goat anti-Mouse IgG Secondary Antibody (Li-Cor Biosciences).

### Cell culture

Human embryonic kidney 293 (HEK293) cells and SW480 cells from American Type Culture Association (ATCC) were cultured in DMEM (Sigma-Aldrich) containing 10% Fetal Bovine Serum (Gibco) and 1% Penicillin-Streptomycin solution (Gibco) at 37°C in 5% CO_2_/95% air.

### siRNA knockdown

Knockdown of various tyrosine kinase targets was achieved using Trilencer-27 Oligo Duplex siRNA (OriGene). Duplexes were resuspended following the manufacturer’s protocol. Transfections with the siRNA were performed using Invitrogen Lipofectamine RNAiMAX Transfection Reagent (Invitrogen) following the manufacturer’s protocol. Initially, the percent protein knockdown of each duplex was determined by Western Blot analysis. After determining the duplex with the most efficient protein knockdown, future experiments were performed using only the most effective duplex.

### Short-term cytotoxicity assay

For imatinib and nilotinib treatments, HEK293 cells were seeded into a 12-well plate at 110,000 cells per well. After allowing to incubate for 24 h at 37°C, the cells were treated at approximately 40% confluency with indicated doses of imatinib or nilotinib as well as a control dose of DMSO. After another 24 h incubation period, the media on the wells was removed and replaced with the following 6 TG doses, one dose per row: NaOH (Control), 5 µM, 10 µM. Cells were then allowed to incubate at 37°C for 48h before being trypsinized and counted *via* cell counter. For siRNA cytotoxicity assays, HEK293 cells were first seeded into a 6-well plate with 600,000 cells per well. After 24 h of incubation at 37°C, cells were transfected with scrambled control or siRNA using the RNAiMax transfection protocol. Cells were placed back in the incubator for another 24 h, then trypsinized, seeded into 12-well plates, treated with 6 TG, and counted following the steps outlined above. Live cell counts were recorded, and percentages were plotted for graphical analysis.

### Cell lysis and collection

Cells were lysed by first removing all media from the plates and washing twice with ice-cold PBS. Cells were lysed using 200–500 µl RIPA lysis buffer (Thermo Scientific) containing either complete protease inhibitor cocktail (Roche) or a combination protease/phosphatase inhibitor cocktail (Cell Signalling). Cellular extracts were collected and stored at −20°C.

### RT-PCR

HEK293 cells were seeded into a 6-well plate with 600,000 cells per well and allowed to incubate for 24 h at 37°C. Cells were then either treated with imatinib or nilotinib or transfected with siRNA. After another 24 h incubation (excluding treatment timecourse experiments), RNA extraction of the cells was performed using the Qiagen RNA Extraction Kit. RNA concentrations were determined using a Nanodrop, then RNA was converted to cDNA using the Superscript cDNA Conversion Kit (Thermofisher). Following the RT-PCR SYBR Green protocol, RT-PCR was set up in a 96-well plate with MLH1 and PCNA primers, with PCNA primers acting as the control. The experiment was run and analyzed using the QuantStudio application and software.

### Nuclear protein extraction

Cells were washed with PBS and resuspended in cytoplasm extract buffer (20 mM HEPES, 10 mM KCl, 0.1 mM EDTA, 1 mM DTT, and protease inhibitor) and then chilled on ice for 10 min 0.75% Nonidet P-40 (NP-40) lysis buffer was added, and the solution was pipetted to mix and vortexed for 10 s. The cells were centrifuged at 800 x g for 3 min at 4°C to separate nuclei from the cytoplasm (supernatant). The cytoplasm extract was placed in a separate tube, and the nuclei pellet was resuspended in 25% sucrose/cytoplasm extraction buffer and pipetted to disperse. The cells in 25% sucrose/cytoplasm extraction buffer were underlaid with half the volume of 50% sucrose/cytoplasm extraction buffer and centrifuged at 10,000 x g for 15 min at 4°C. The supernatant was removed, and the nuclei pellet was lysed in PBE150Na [50 mM Tris-HCl at pH 7.5, 1 mM ethylenediaminetetraacetic acid (EDTA) at pH 8.0, 150 mM NaCl, 0.5% sodium deoxycholate and 1% NP-40, containing 1x Complete protease inhibitor cocktail (Roche Diagnostics GmbH, Germany)]. The pellet was then sonicated and centrifuged at 10,000x g for 15 min at 4°C. The supernatant was collected as the nuclear extract.

### Immunoprecipitation

Co-immunoprecipitations of endogenous proteins were performed using magnetic protein A/protein G beads (Thermo Scientific), followed by a conjugation step to either the IgG control or antibody of interest. Beads were blocked with BSA for 1 h, followed by washes. Conjugated beads were incubated with samples at 4°C for 3 h rotating, followed by increasing salt washes. Beads were boiled with 6x loading buffer, and samples were run on SDS-PAGE gels followed by Western blot.

### Kinase assay

Kinase assays were performed using recombinant ABL1 (ABL1) (25ng, Invitrogen P3049) and MLH1 (100ng, Origene TP301607) with the addition of kinase buffer (20 mM Tris pH 7.4, 10mM MgCl_2_, 1 mM DTT) and 1 mM cold ATP. Each reaction was assembled in separate 1.5 ml microcentrifuge tubes and placed in a heat block at 37°C. The reaction was quenched by adding 4x Laemeli buffer and heating at 95°C for 5 min. The samples were then analyzed by Western blot. Initially, we performed a timecourse and quenched the reaction at the following time points after the addition of ATP: 0, 15, 30, and 60 min. We determined that 15 min after ATP addition was sufficient enough to observe tyrosine phosphorylation (Data not shown).

## Quantification and statistics

Immunoblots were imaged using LiCor infrared secondary antibodies and a LiCor Odyssey Infrared Western Blot Imaging system. Band intensity was calculated using the LiCor software and normalized to the band intensity of the loading control. Calculations of the mean, standard error, statistical analysis, and comparison of each set of experimental means were performed with Graphpad Prism 9.0 (Graphpad Software Inc., La Jolla, CA, United States). Comparison between conditions was performed with either an unpaired t-test or 1-way Anova.

## Results

### Treatment with receptor tyrosine kinase inhibitor, dasatinib, in SW480 cells decreases MLH1 protein expression

**FIGURE 1:**
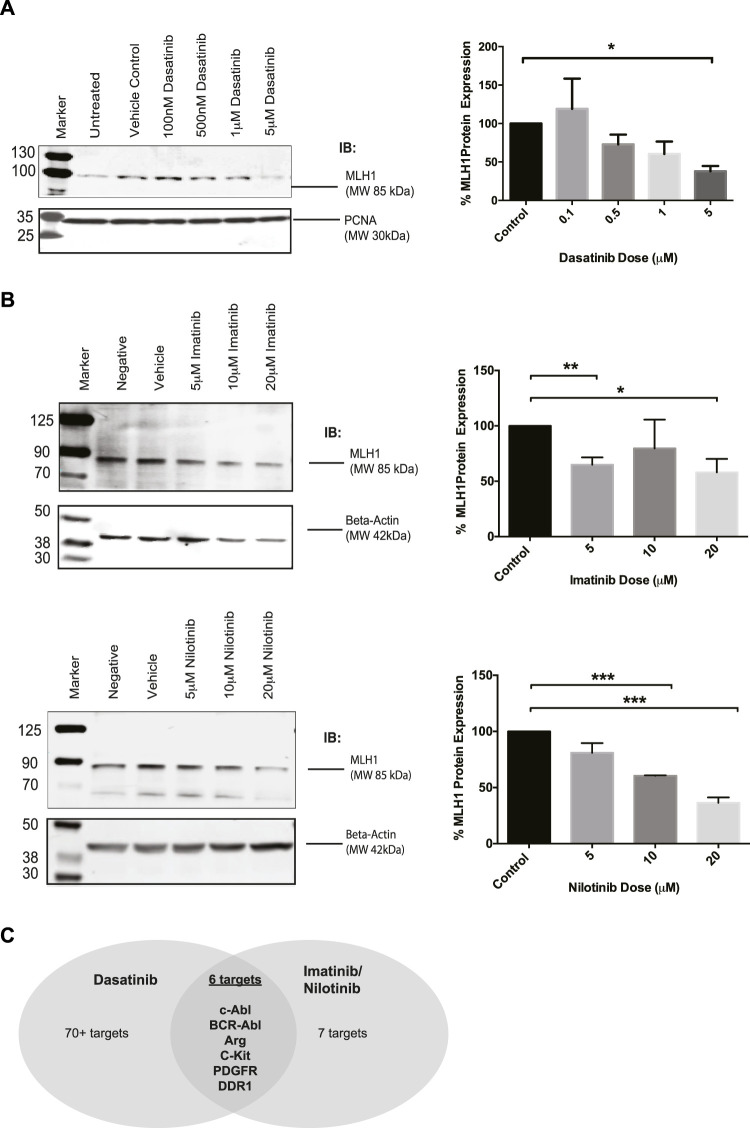
MLH1 protein levels are reduced in SW480 colorectal cells after treatment with Dasatinib and imatinib/nilotnib tyrosine kinase inhibitors. **(A)** Western blot analysis and quantification of MLH1 protein expression after treatment with Dasatinib in SW480 colorectal cancer cells *n* = 3 statistical significance was determined by unpaired t-test*p < 0.05 **(B)** Western blot analysis and quantification of MLH1 protein expression after treatment with imatinib (top) and nilotinib (bottom) in SW480 colorectal cancer cells *n* = 3 statistical significance was determined by 1-way ANOVA with Bonferroni post-test *n* = 3 *p < 0.05, **p < 0.01, ***p < 0.001 **(C)** Target comparison of Dasatinib versus imatinib/nilotinib based on (Hantschel et al., 2008).

There is interest in investigating SRC-kinase inhibitors in advanced colorectal cancer patients in combination with other chemotherapy (Parseghian et al., 2017; Scott et al., 2017). During investigations into the relationship between MMR status and response to tyrosine kinase inhibitors, we unexpectedly observed that Dasatinib-treated cells had reduced levels of MLH1 protein. In MMR proficient SW480 colorectal cells treated with increasing doses of Dasatinib, MLH1 expression was decreased in a dose-dependent manner, with a statistically significant effect seen at 5 μM Dasatinib, resulting in roughly 50% MLH1 expression remaining. (Figure 1A).

Dasatinib has upwards of 70 known targets (Hantschel et al., 2008). With the understanding that Dasatinib is a third-generation SRC-kinase inhibitor with a wide range of targets, we decided to use earlier generation drugs more commonly used in the clinic, imatinib and nilotinib, to narrow down the kinase target responsible for the decrease in MLH1 protein expression. Imatinib and nilotinib have the same seven known targets (Hantschel et al., 2008). We treated SW480 cells with either imatinib or nilotinib and confirmed that MLH1 protein expression was decreased in a similar manner to Dasatinib treatment (Figure 1B). When comparing the targets between Dasatinib and imatinib/nilotinib, there are six common target kinases: Kit, PDGFR, ABL1, DDR1, ARG (ABL2), and the fusion kinase present only in certain leukemias, BCR-Abl (Hantschel et al., 2008) (Figure 1C). Based on our observations, we believed the kinase target responsible for the decrease in MLH1 protein expression was common between all three tyrosine kinase inhibitors.

### Imatinib and nilotinib treatment decreases MLH1 protein expression and inhibits mismatch repair-mediated apoptosis

**FIGURE 2:**
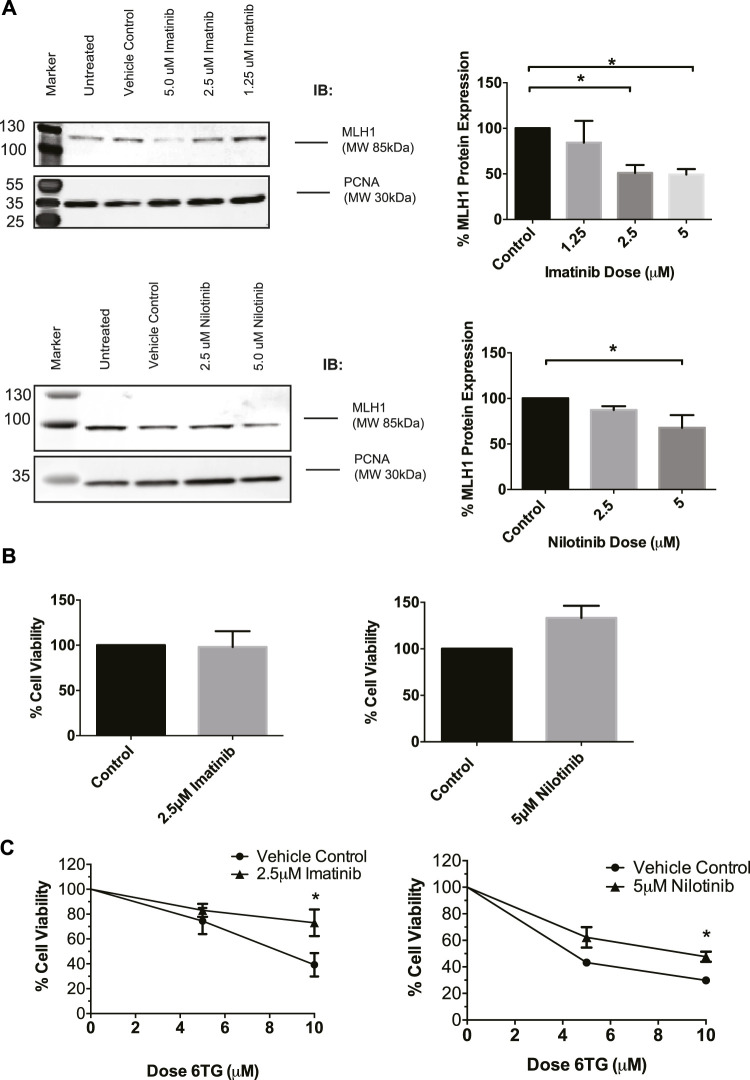
MLH1 protein levels are decreased and MMR function inhibited in HEK293 cells after treatment with tyrosine kinase inhibitors. **(A)** Western blot analysis and quantification of MLH1 protein expression after treatment with imatinib (top) and nilotinib (bottom) in HEK293 cells statistical significance determined by 1-way ANOVA with Bonferroni post-test *n* = 3 *p < 0.05 **(B)** Treatment with imatinib or nilotinib alone does not significantly affect cell viability in HEK293 cells measured *via* cell count 24 h after treatment **(C)** Cell survival measured *via* viable cell count after 48-h co-treatment with imatinib/nilotinib and 6TG, statistical significance was determined by unpaired t-test *n* = 3 *p < 0.05.

To determine if this was a common phenomenon or specific to cancer cells, we used HEK293 cells going forward due to their proficient MMR mechanism and lack of cancer-specific alterations. To confirm that imatinib and nilotinib have a similar effect on MLH1 protein expression, HEK293 cells were treated with a varying dose range of each drug, respectively. Results show that imatinib and nilotinib treatment decrease MLH1 protein expression in HEK293 cells, similarly to what was observed in SW480 cells. (Figure 2A). We looked at other MMR proteins, including MSH2, MSH6, and Exo1, after nilotinib treatment in HEK293 cells and determined the effect of the drug to primarily be on MLH1 protein expression (Supplementary Figure 1A). We also observed changes to the MLH1 obligate binding partner, PMS2, after nilotinib treatment (Supplementary Figure 1B).

To determine if the approximately 50% reduction in MLH1 levels was sufficient to affect the efficiency of the MMR pathway, cell viability after imatinib or nilotinib treatment in combination with 6 thioguanine (6TG) was examined. 6TG is a purine antimetabolite that is incorporated into the DNA during replication, ultimately leading to an O^6^ methylguanine mismatch recognized by MMR. The mispair cannot be repaired, resulting in apoptosis when MMR is functional and resistance to 6TG when MMR is defective (Meyers et al., 2004; O'Brien and Brown, 2006). Loss of MLH1 protein results in resistance to 6TG due to loss of MMR-induced apoptotic signaling in response to the O^6^-T mispair (WE et al., 1998; Niv, 2007; Zhao et al., 2019). We determined that imatinib or nilotinib treatment alone did not change cell viability (Figure 2B). However, in a 72-h survival assay, a 24-h pre-treatment with imatinib or nilotinib decreased cell death after 6 TG treatment compared to cells pretreated with the vehicle control (Figure 2C). Based on these data, we suggest that treatment with tyrosine kinase inhibitors leads to an impaired MMR damage response by the downregulation of MLH1 in a variety of non-cancerous and cancerous cell lines.

### ABL1 knockdown decreases MLH1 expression

**FIGURE 3:**
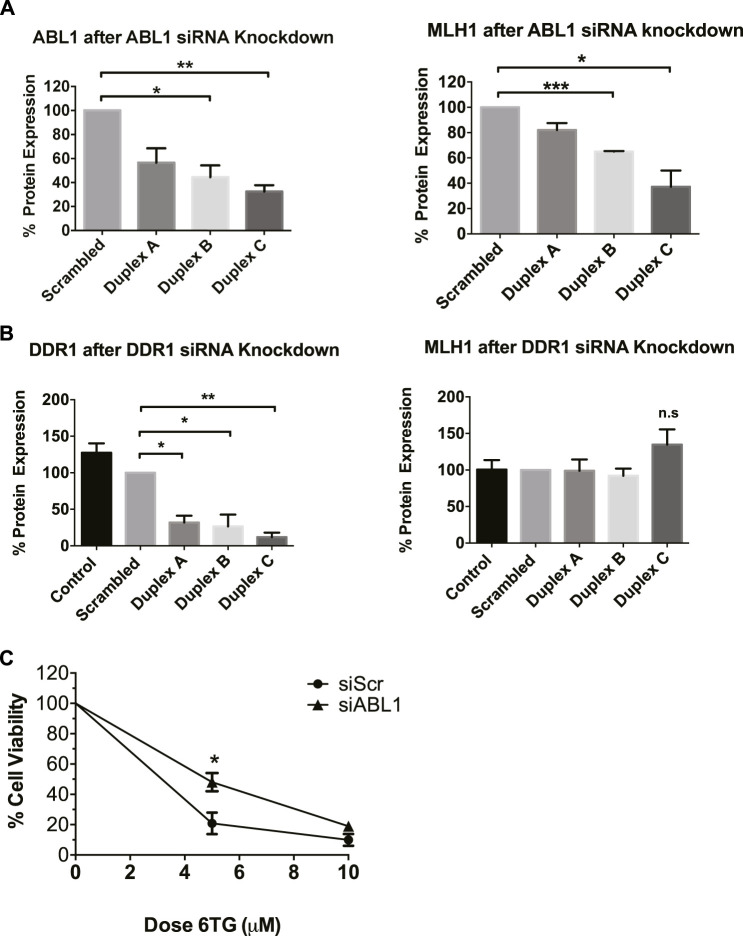
ABL1 but not DDR1 knockdown reduces MLH1 protein level and function. **(A)** Quantification of western blots for ABL1 (left) and MLH1 (right) after transfection with siRNA duplexes against ABL1 or scrambled control in HEK293 cells. *n* = 3, statistical significance determined by 1-way ANOVA with Bonferroni post-test *p < 0.05 **(B)** Quantification of western blots for DDR1 (left) and MLH1 (right) after transfection with siRNA duplexes against DDR1 or scrambled control in HEK293 cells. *n* = 3, statistical significance determined by 1-way ANOVA with Bonferroni post-test *p < 0.05. **(C)** Cell survival measured *via* viable cell count after treatment with 6TG after siRNA knockdown of ABL1 using duplex C, *n* = 3 significance was determined by unpaired t-test *p < 0.05.

After determining that imatinib and nilotinib treatment impairs MMR-mediated apoptosis, we questioned which of the known kinase targets were responsible. There are seven known targets of imatinib and nilotinib: BCR/ABL, c-Kit, DDR1, PDGFRα, ABL1 (ABL1), ARG (ABL2), and NQO1 (Hantschel et al., 2008). BCR/ABL is a fusion kinase only present in certain leukemia cell lines, not HEK293 cells, leaving six targets of interest. Of these six targets, ABL1 specifically has been suggested to physically interact with MLH1 (Wagner et al., 2008; Fukuhara et al., 2014; Li et al., 2018). Based on this, we obtained siRNA duplexes for ABL1 and a target kinase not implicated in MMR, discoidin domain receptor tyrosine kinase 1, DDR1. HEK293 cells were transfected with each siRNA duplex separately, and Western blot analysis was performed to determine knockdown efficiency and changes to MLH1 protein expression. Knockdown of ABL1 resulted in a corresponding decrease in MLH1 protein expression levels similar to that seen with imatinib or nilotinib treatment (Figure 3A, Supplementary Figure 2A). In contrast, knockdown of DDR1 resulted in no significant change to MLH1 protein expression (Figure 3B; Supplementary Figure 2B).

To confirm that decreased MLH1 protein expression by ABL1 knockdown also affected MMR-mediated apoptosis, we performed the short-term cytotoxicity assay with 6TG treatment in HEK293 cells with either ABL1 knockdown or scrambled siRNA control. ABL1 knockdown increased resistance to MMR-mediated apoptosis after 6TG treatment to a similar level as that seen with nilotinib or imatinib (Figure 3C).

To determine the level of ABL1 activity in the SW480 and HEK293 cells and to confirm that imatinib/nilotinib treatment effectively inhibited ABL1 activity in these cells, we tested phosphorylation of Crk-like protein (CrkL). CrkL is phosphorylated by ABL1 and can be considered a surrogate marker of overall ABL1 activity (Ganguly et al., 2012; Jain et al., 2017; Tripathi et al., 2020). We determined that the SW480 cell line has increased ABL1 activity compared to HEK293 cells and that imatinib treatment does decrease pCrkL expression, however, only partially in the HEK293 cells (Supplementary Figure 3A). On the other hand, nilotinib treatment in HEK293 cells resulted in almost complete abolishment of ABL1 activity, indicated by the lack of pCrkL protein expression (Supplementary Figure 3B). We conclude that the ABL1 kinase is likely the imatinib/nilotinib target kinase responsible for decreased MLH1 protein expression and the subsequent impairment of MMR.

### MLH1 downregulation by ABL1 inhibition is posttranslational and involves lysosomal degradation

**FIGURE 4:**
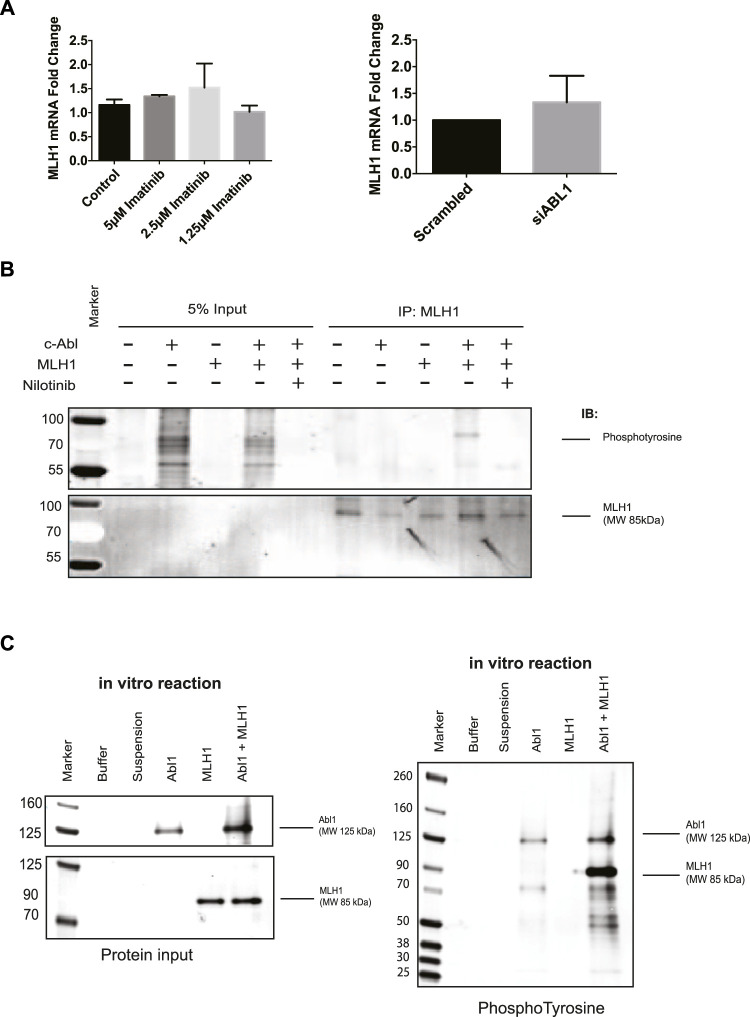
ABL1 phosphorylates MLH1. **(A)** MLH1 mRNA fold change with ABL1 knockdown (left) or inhibition by imatinib (right) determined by RT-qPCR *n* = 3 **(B)** MLH1 immunoprecipitation in HEK293 cells with ABL1 and MLH1 overexpressed followed by immunoblot with anti-phosphotyrosine antibody. Treatment with 5 μM nilotinib reduces the phospho-tyrosine signal associated with MLH1. **(C)** Kinase assay using recombinant ABL1 (25 ng) and MLH1 (100 ng) protein incubated for 15 min, followed by SDS-PAGE and immunoblot analysis indicates tyrosine phosphorylation of MLH1 only in the presence of ABL1 kinase.

The MLH1 protein is highly stable (>24 h) (Supplementary Figure 3C), and while it is well known that MLH1 expression can be controlled by promotor methylation, there are few studies looking into its degradation (Kane et al., 1997; Hinrichsen et al., 2017a; Abildgaard et al., 2019). To determine the mechanism of MLH1 downregulation after ABL1 inhibition or loss, we first investigated any changes to mRNA levels by RT-PCR. We observed no significant mRNA change between control or treated samples in ABL1 knockdown or inhibitor conditions at the 24-h timepoint at which protein loss is observed (Figure 4A). No mRNA change is observed after nilotinib treatment at any prior timepoint either (Supplementary Figure 4A).

Based on these results, we shifted our focus to mechanisms of posttranslational downregulation, starting with the potential tyrosine phosphorylation of MLH1 by ABL1. The ABL1 kinase phosphorylates various proteins on tyrosine residues for protein activation, protein degradation, or protein stability (Wang, 1993; Yuan et al., 1996; Shaul, 2000). To determine if MLH1 is phosphorylated by ABL1, ABL1, and MLH1 were overexpressed in HEK293 cells in the presence or absence of nilotinib, followed by immunoprecipitation of MLH1 and immunoblot using a pan-phosphotyrosine antibody. Western blot analysis showed a phosphotyrosine band corresponding to the size of MLH1 in the ABL1 and MLH1 overexpressed sample that was no longer detectable after ABL1 inhibition by nilotinib (Figure 4B). These results indicate that ABL1 tyrosine phosphorylates MLH1, and this phosphorylation is blocked after ABL1 inhibition. Thus, ABL1-mediated phosphorylation of MLH1 may prevent MLH1 degradation. To determine if this was a direct phosphorylation event by ABL1, recombinant ABL1 (ABL1) and MLH1 proteins were incubated with ATP in an *in vitro* kinase reaction. The reaction was run on an SDS-PAGE gel and probed with an anti-phosphotyrosine antibody. A phosphorylated band was observed at the 85 KDa molecular weight corresponding to MLH1 only when incubated with ABL1 protein (Figure 4C). Together, we conclude that ABL1 directly phosphorylates MLH1 on a tyrosine residue (Figure 4C).

**FIGURE 5:**
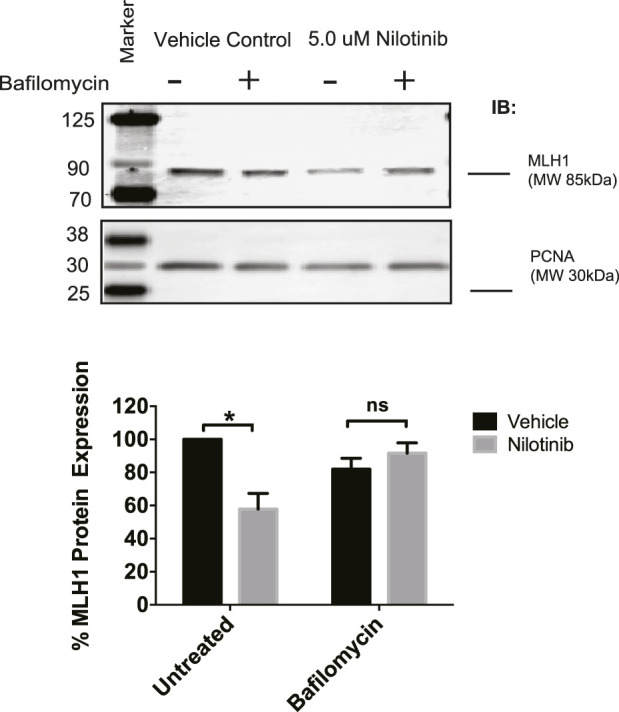
Inhibition of lysosomal degradation restores MLH1 levels reduced by nilotinib treatment. Western blot analysis and quantification of MLH1 protein expression after nilotinib or vehicle control co-treated with 100 nM Bafilomycin or vehicle control, *n* = 3 significance was determined by unpaired t-test *p < 0.05.

Next, we determined whether the observed loss of MLH1 after ABL1 inhibition occurs *via* lysosomal or proteasomal degradation pathways. HEK293 cells were co-treated with nilotinib and either Bafilomycin or MG-132 to inhibit lysosomal or proteasomal degradation, respectively. We observed that when cells were co-treated with Bafilomycin and nilotinib for 24 h, there was a rescue of MLH1 protein expression levels compared to cells treated with nilotinib alone (Figure 5). In contrast, the proteasomal inhibition by MG-132 did not prevent MLH1 downregulation after nilotinib treatment (Supplementary Figure 4B). We also observed no ubiquitination of MLH1 when MLH1 and HA-tagged ubiquitin were co-expressed, and MLH1 was immunoprecipitated and then probed with the anti-HA antibody (Supplementary Figure 4C).

### Hsp70 is a chaperone for MLH1 lysosomal degradation when ABL1 is inhibited

**FIGURE 6:**
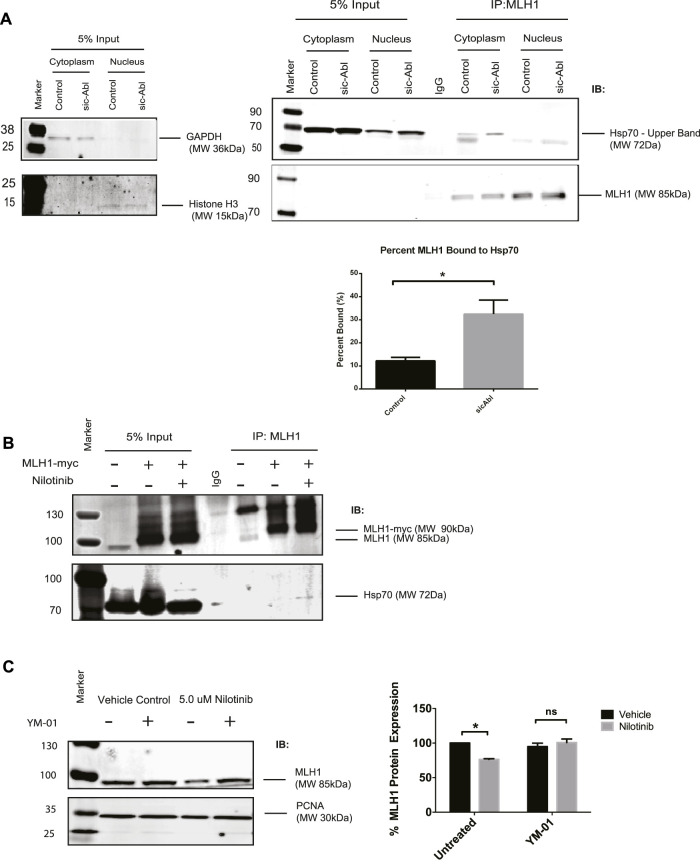
MLH1 binds to Hsp70 in the absence of ABL1 signaling and inhibition of Hsp70 restores MLH1 levels. **(A)** MLH1 immunoprecipitation from nuclear or cytoplasmic fractions in cells transfected with scrambled control or ABL1 siRNA followed by immunoblot for Hsp70 and MLH1. Cytoplasmic and nuclear fractionation purity was confirmed by GAPDH and Histone H3 immunoblots, respectively. Quantification of percent MLH1 bound to Hsp70 is shown. *n* = 3, significance was determined by unpaired t-test *p < 0.05. **(B)** MLH1 immunoprecipitation from whole cell lysate of HEK293 cells overexpressing MLH1 and treated with nilotinib or vehicle control, followed by immunoblot for Hsp70 and MLH1 **(C)** Immunoblot analysis and quantification of MLH1 protein expression in HEK293 cells after treatment with nilotinib or vehicle control after 24 h of co-treatment with 1 μM Hsp70 inhibitor, YM-01, or vehicle control. *n* = 3, significance was determined by unpaired t-test *p < 0.05.

A recent study showed that MLH1 mutant variants predicted by computational modeling to be unstable were more rapidly degraded by the proteasome than WT MLH1, presumable due to cellular unfolded protein response. The authors also showed that these unstable variants of MLH1 had increased interaction with the Hsp70 chaperone protein (Abildgaard et al., 2019). We hypothesized that Hsp70 may be a universal chaperone for MLH1 and tested whether Hsp70 was required for MLH1 degradation in the absence of ABL1 activity. We first examined whether Hsp70 interaction with MLH1 is dependent on ABL1. Using subcellular fractionation followed by immunoprecipitation of endogenous MLH1 and immunoblotting for Hsp70, we found that MLH1 and Hsp70 interact in the cytoplasm, and this interaction was significantly increased after silencing of ABL1 (Figure 6A). We also overexpressed MYC-FLAG-tagged MLH1 and treated cells with nilotinib, followed by immunoprecipitation of MLH1 and immunoblot for Hsp70. Like ABL1 knockdown, treatment with nilotinib increased Hsp70 interaction with MLH1 (Figure 6B). To determine whether binding to Hsp70 had an impact on MLH1 degradation, we co-treated cells with nilotinib and an Hsp70 inhibitor, YM-01. YM-01 treatment rescued the reduction in MLH1 protein expression induced by nilotinib treatment (Figure 6C). Taken together, we propose that ABL1 is important for maintaining MLH1 protein stability in at least a subset of cellular MLH1 pools, potentially by phosphorylation of MLH1. In the absence of ABL1, MLH1 is targeted by Hsp70 for lysosomal degradation.

## Discussion

This study identifies a novel mechanism of MLH1 regulation through the involvement of the ABL1 kinase, Hsp70, and lysosomal degradation. We first observed changes in MLH1 protein expression levels after treatment with the FDA-approved tyrosine-kinase inhibitor, Dasatinib. Since this inhibitor targets a large number of kinases, we systematically narrowed down the target kinase responsible by using tyrosine-kinase inhibitors with a narrower range of targets, imatinib and nilotinib, and siRNA to knock down specific kinases. ABL1 has previously been reported to physically interact with MLH1 (Kim et al., 2007), so we chose to focus on that target kinase first. We show here that ABL1 kinase knockdown by siRNA recapitulates the MLH1 down-regulation, MMR impairment, and Hsp70 binding that we observe with the nilotinib or Dasatinib treatment. We also tested DDR1 siRNA as a comparison for a protein not known to impact MMR, and we did not observe any MLH1 protein change. HEK293 cells do not have appreciable levels of PDGFR or c-Kit (Zaslavsky et al., 2005; Nakagomi and Hirota, 2007), so it is unlikely these contribute to the MLH1 degradation observed. While the degree of MLH1 protein reduction was roughly equivalent between pharmacological inhibition and siRNA knockdown of ABL1 in HEK293 cells, we cannot rule out the possibility that either c-Kit or PDGFR may influence MMR in specific tumor backgrounds where they are highly expressed or mutated. We did not evaluate the role of NQO1 as it is a quinone reductase instead of a non-receptor tyrosine kinase (Hantschel et al., 2008). The fusion protein BCR-ABL is produced by the Philadelphia chromosome translocation found in leukemia. This fusion produces a dysregulated constitutively active form of ABL1. Given our findings here that normal ABL1 activity is important for MLH1 protein stability in at least a portion of cellular MLH1, it raises interesting questions as to what MLH1 dynamics are in leukemia cells harboring BCR-ABL. It also raises questions about MMR function in solid tumors with hyperactive ABL1, such as subsets of melanoma or triple-negative breast cancer (Srinivasan and Plattner, 2006; Srinivasan et al., 2008; Ganguly et al., 2012; Ganguly and Plattner, 2012). Skorski et al. report that the Leukemia cells with the BCR-ABL fusion also have dysfunctional MMR, including increased mutation frequencies and decreased sensitivity to alkylating agents (Stoklosa et al., 2008). This report, together with our findings, suggests a balance of ABL1 activity is required for MMR, with both too little or too much activation resulting in MMR deficiency, although potentially through different mechanisms.

Here, we identified one MMR protein whose stability is controlled by ABL1 activity. However, there are likely other MMR proteins whose activity or stability is controlled by kinase activity. Li et al. published a series of papers in which they showed that PCNA is phosphorylated on tyrosine residue 211 by EGFR (Ortega et al., 2015). This phosphorylation can be induced by arsenic exposure (Tong et al., 2015). Phosphorylation of PCNA prevented MMR activity by altering the interaction of PCNA with critical MMR proteins (Ortega et al., 2015). Bardelli et al. showed that targeting EGFR/BRAF downregulates MMR activity (Russo et al., 2019). However, treatment with either EGFR inhibitor alone or EGFR inhibitor with BRAF inhibitor decreased numerous repair pathway proteins, including those involved in homologous recombination and base excision repair. MMR was downregulated across most of the critical protein components, including MLH1, PMS2, MSH2, and MSH6. In this case, the regulation appears to be at the mRNA level and corresponds with mRNA upregulation of error tolerance pathways such as translesion polymerases (Russo et al., 2019). We observe protein downregulation but not mRNA downregulation after ABL1 inhibition by either siRNA or tyrosine kinase inhibitors. In our study, we also observe a concurrent loss of PMS2 protein levels but not a loss of MSH2 and MSH6. PMS2 is an obligate dimer with MLH1, and loss of MLH1 protein leads to loss of PMS2 protein (Wu et al., 2003; Kosinski et al., 2010). We focused on MLH1 in this study as MLH1 is the common partner for the MutL heterodimers. MLH1 dimerizes with MLH2, MLH3, and PMS2, although MLH1-PMS2 plays the most prominent role in MMR (Wang et al., 1999; Campbell et al., 2014). We observe phosphorylation of MLH1 after immunoprecipitation and immunoblot with a pan-phosphotyrosine antibody. We also observed phosphorylation of MLH1 by ABL1 in an *in vitro* kinase assay with purified recombinant ABL1 and MLH1, confirming there is a direct phosphorylation between ABL1 and the MLH1 component of the MLH1-PMS2 heterodimer.

While the effect on MLH1 was consistent with ABL1 inhibition or knockdown, it consistently only lowered MLH1 levels by 25%–50%, even when ABL1 was knocked down to about 30% expression. Phosphorylation by overexpressed ABL1 in HEK293 cells also appears only to affect a moderate portion of the pulled-down MLH1 (Figure 4). The cellular conditions or the specific cellular pool of MLH1 in which ABL1 controls MLH1 expression are still unknown. MLH1 is primarily nuclear but has some minor cytoplasmic expression and is imported by a nuclear localization sequence (Wu et al., 2003). ABL1 has expression in both the nucleus and the cytoplasm. ABL2, also known as ARG, shares many roles with ABL1 but has some distinct roles and is only cytoplasmic (Ganguly et al., 2012). ABL2 is commonly also targeted by imatinib/nilotinib; thus, we cannot rule out ABL2 as also influencing MLH1. The potential impact of ABL2 in addition to ABL1 is currently under investigation.

Utilizing drugs Bafilomycin and MG-132 to inhibit lysosomal or proteasomal degradation, respectively, we found that by inhibiting lysosomal degradation by Bafilomycin, MLH1 protein expression loss was prevented in the presence of ABL1 inhibition. Findings from a recent computational study led us to investigate the potential involvement of chaperone protein, Hsp70 (Abildgaard et al., 2019). Though Hsp70 is mainly reported to be involved in protein refolding, it has also been shown to be involved in presenting proteins for degradation by autophagy (Fernandez-Fernandez et al., 2017). Immunoprecipitation results showed increased interaction between a subset of the MLH1 cellular pool and Hsp70 after ABL1 inhibition. After inhibition of Hsp70 by pharmacological inhibitor YM-01, MLH1 protein expression loss was completely prevented in cells with ABL1 inhibition. In combination with previous studies, these data suggest that Hsp70 may act as a chaperone for MLH1 degradation in general.

Overall, this study demonstrates a previously uncharacterized regulatory pathway of MLH1 by ABL1 and suggests that phosphorylation of MLH1 is required for its protein stability. ABL1 inhibition causes a loss of phosphorylation in at least a subset of cellular MLH1, leading to Hsp70 chaperone binding and lysosomal degradation. These data have interesting and important clinical implications given the rise of immunotherapy use in MSI-high/MMR deficient tumors (Kubecek et al., 2016). These data may present a strategy for sensitizing MSI-low/MMR proficient tumors to immunotherapy, especially as tyrosine kinase inhibitors are FDA-approved drugs with a known safety profile.

## Data Availability

The raw data supporting the conclusion of this article will be made available by the authors, without undue reservation.
