# Supplementary figures and images for: Inhibition of ABL1 by tyrosine kinase inhibitors leads to a downregulation of MLH1 by Hsp70-mediated lysosomal protein degradation
Source: Front Genet. 2022 Oct 20;13:940073. doi: 10.3389/fgene.2022.940073 (PMC9631443; doi:10.3389/fgene.2022.940073)

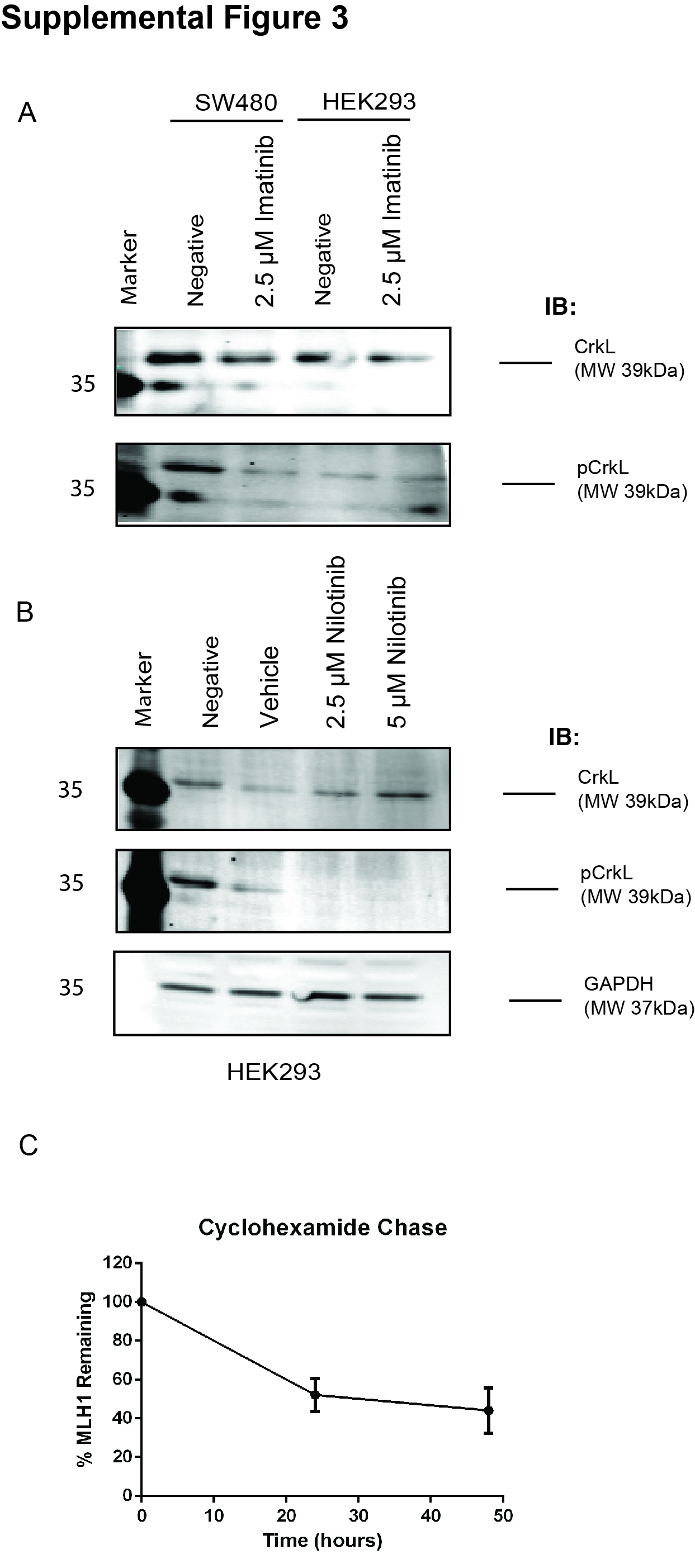

Supplement: Supplementary file 1 [file Image3.JPEG]

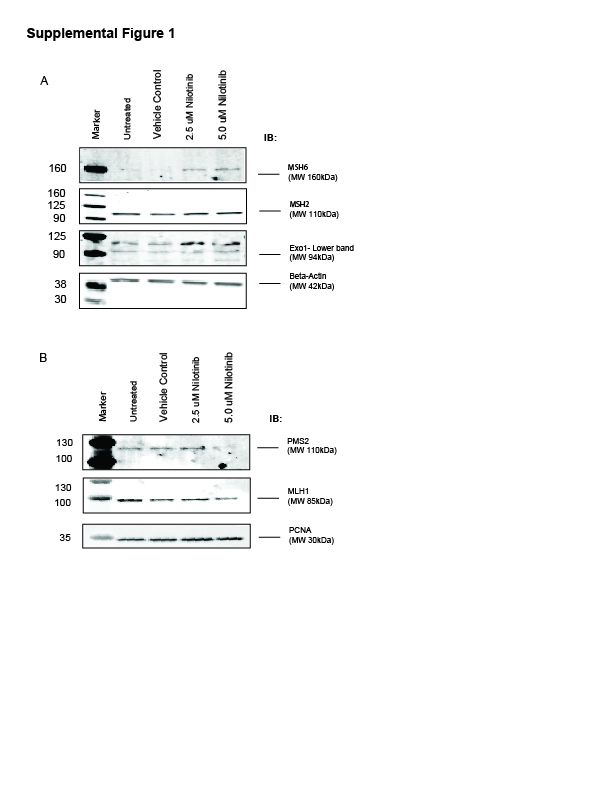

Supplement: Supplementary file 3 [file Image1.JPEG]

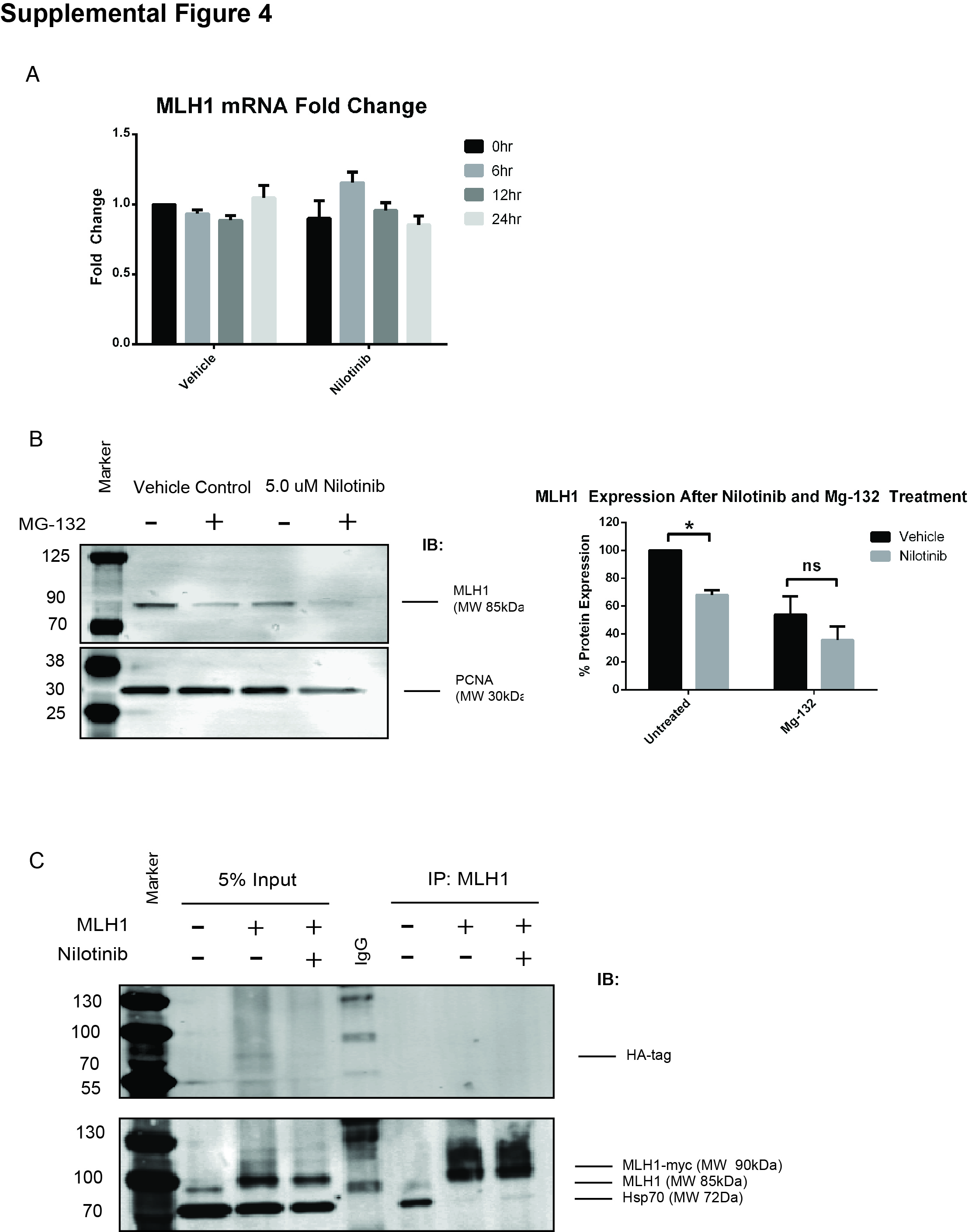

Supplement: Supplementary file 4 [file Image4.JPEG]

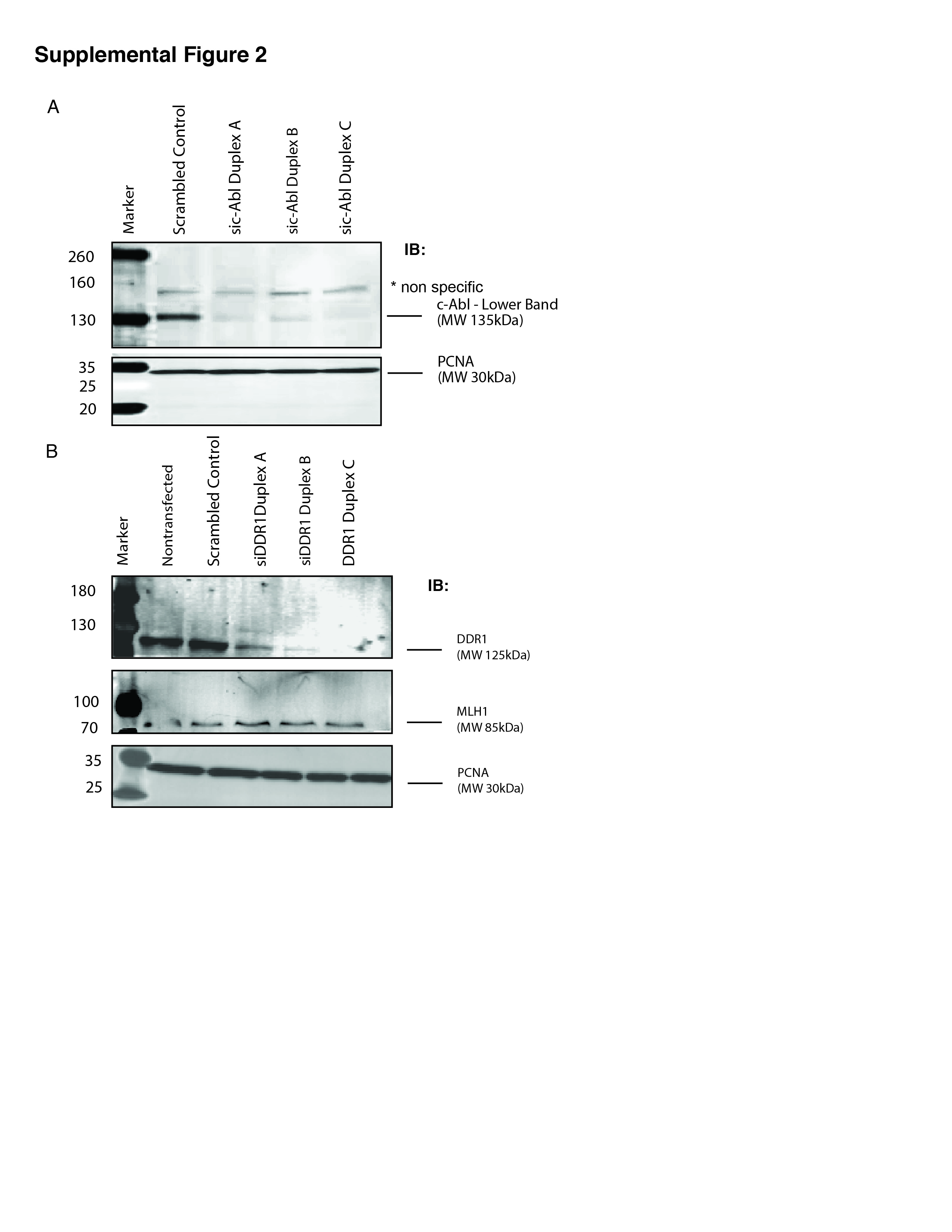

Supplement: Supplementary file 5 [file Image2.JPEG]
