# Supplementary material for: Inhibition of ABL1 by tyrosine kinase inhibitors leads to a downregulation of MLH1 by Hsp70-mediated lysosomal protein degradation
Source: Front Genet. 2022 Oct 20;13:940073. doi: 10.3389/fgene.2022.940073 (PMC9631443; doi:10.3389/fgene.2022.940073)
Supplement: Supplementary file 2 [file Table1.DOCX]

**Supplemental Figure 1**: **A)** Western blot analysis of other critical MMR proteins after nilotinib treatment including MSH6, MSH2, and Exo1 in HEK293 cells. No other protein was shown to significantly decrease after nilotinib treatment. **B)** Western blot analysis of PMS2 in HEK293 cells treated with Nilotinib. PMS2 is an obligate heterodimer with MLH1 and nilotinib also induced a decrease in PMS2.

**Supplemental Figure 2:** Representative western blot images corresponding to quantification shown in main text Figure 3 of **A)** ABL1 and MLH1 protein levels after siRNA knockdown of ABL1 with 3 independent siRNA duplexes in HEK293 cells. **B)** DDR1 and MLH1 protein levels after siRNA knockdown of DDR1 with 3 independent siRNA duplexes in HEK293 cells.

**Supplemental Figure 3:** **A)** Western blot comparison of CrkL and pCrkL levels before and after imatinib treatment in SW480 and HEK293 cells. **B)** Western blot comparison of CrkL and pCrkL levels before and after nilotinib treatment in HEK293 cells. **C)** Western blot quantification of cycloheximide chase experiments to determine stability of native MLH1 over a 48-hour time period.

**Supplemental Figure 4: A)** MLH1 mRNA fold change before and after 5μM Nilotinib treatment at various time points; 0hr, 6hr, 12hr, and 24hr. No significant changes to the MLH1 mRNA fold change were observed at any given time point. **B**) Western blot analysis and quantification of MLH1 protein expression after Nilotinib or vehicle control treated with 1 μM MG-132 or vehicle control 4 hours before cell collection N=3 significance was determined by unpaired t-test *p<0.05 **C)** MLH1 was immunoprecipitated from HEK293 cells transfected with myc-Flag tagged MLH1 and HA-tagged ubiquitin and treated with Nilotinib or vehicle control. Blots were probed with anti-HA, anti-MLH1, and anti-Hsp70 antibodies. No ubiquitin associated with MLH1 was observed. Hsp70 was also detected as co-immunoprecipitating with MLH1 and the interaction increased with Nilotinib treatment as observed in main Figure 7.
